# Grey matter volumetric changes related to recovery from hand paresis after cortical sensorimotor stroke

**DOI:** 10.1007/s00429-014-0804-y

**Published:** 2014-06-07

**Authors:** E. Abela, A. Seiler, J. H. Missimer, A. Federspiel, C. W. Hess, M. Sturzenegger, B. J. Weder, R. Wiest

**Affiliations:** 1 Support Center for Advanced Neuroimaging (SCAN), Institute for Diagnostic and Interventional Neuroradiology, University Hospital Inselspital and University of Bern, Bern, Switzerland; 2 Department of Neurology, Kantonsspital St. Gallen, St. Gallen, Switzerland; 3 Department of Neurology, University Hospital Inselspital and University of Bern, Bern, Switzerland; 4 Laboratory of Biomolecular Research, Paul Scherrer Institute, Villigen, Switzerland; 5 Department of Psychiatric Neurophysiology, University Hospital of Psychiatry and University of Bern, Bern, Switzerland

**Keywords:** Cortical stroke, Grey matter plasticity, Tensor-based morphometry, Motor recovery

## Abstract

Preclinical studies using animal models have shown that grey matter plasticity in both perilesional and distant neural networks contributes to behavioural recovery of sensorimotor functions after ischaemic cortical stroke. Whether such morphological changes can be detected after human cortical stroke is not yet known, but this would be essential to better understand post-stroke brain architecture and its impact on recovery. Using serial behavioural and high-resolution magnetic resonance imaging (MRI) measurements, we tracked recovery of dexterous hand function in 28 patients with ischaemic stroke involving the primary sensorimotor cortices. We were able to classify three recovery subgroups (fast, slow, and poor) using response feature analysis of individual recovery curves. To detect areas with significant longitudinal grey matter volume (GMV) change, we performed tensor-based morphometry of MRI data acquired in the subacute phase, i.e. after the stage compromised by acute oedema and inflammation. We found significant GMV expansion in the perilesional premotor cortex, ipsilesional mediodorsal thalamus, and caudate nucleus, and GMV contraction in the contralesional cerebellum. According to an interaction model, patients with fast recovery had more perilesional than subcortical expansion, whereas the contrary was true for patients with impaired recovery. Also, there were significant voxel-wise correlations between motor performance and ipsilesional GMV contraction in the posterior parietal lobes and expansion in dorsolateral prefrontal cortex. In sum, perilesional GMV expansion is associated with successful recovery after cortical stroke, possibly reflecting the restructuring of local cortical networks. Distant changes within the prefrontal-striato-thalamic network are related to impaired recovery, probably indicating higher demands on cognitive control of motor behaviour.

**Electronic supplementary material:**

The online version of this article (doi:10.1007/s00429-014-0804-y) contains supplementary material, which is available to authorized users.

## Introduction

A significant proportion of stroke survivors suffer from long-term sensorimotor deficits of the contralesional arm and hand, notably loss of force and fine motor control (Go et al. 2013). Spontaneous recovery of upper limb motor function occurs during the first few months after ischaemic stroke, usually with highly heterogeneous time courses that are difficult to predict in individual patients (Prabhakaran et al. 2008; Stinear 2010). Understanding the structural and functional neurobiological basis of this process and how it influences variability in individual outcomes might be important for prognostication and design of future restorative therapies (Krakauer 2005; Nudo 2003; Stinear et al. 2012).

In preclinical models, upper limb function recovery after focal ischaemic lesions to the primary sensorimotor cortices (SM1) is paralleled by profound plastic changes in grey matter morphology, including synaptogenesis in perilesional areas, axonal sprouting between the perilesional and premotor cortices (PMC), reorganization of cortical somatotopic sensorimotor representations, and increased dendritic density in the ipsi- and contralesional basal ganglia (Carmichael et al. 2001; Dancause et al. 2005; McNeill et al. 2003; Napieralski et al. 1996; Winship and Murphy 2008). Re-injury to reorganized areas can lead to reappearance of the original deficit, indicating that grey matter plasticity is causally linked to restoration of motor behaviour (Zeiler et al. 2013). Of note, recent magnetic resonance imaging (MRI)-based morphometric studies suggest that changes in cortical and subcortical grey matter volume (GMV) and cortical thickness can be detected in human patients with striato-capsular stroke (Brodtmann et al. 2012; Fan et al. 2012; Schaechter et al. 2006; Schaechter and Perdue 2008). However, the morphological effects of ischaemic cortical lesions in human SM1 on perilesional and remote GMV and their possible relationship to post-stroke recovery have, to the best of our knowledge, not been investigated so far. This might provide insights into post-stroke brain architecture, as a basis to better understand the effect of targeted neurorehabilitative or pharmacological interventions (Brown et al. 2008).

We here therefore analyse structural neuroimaging data from a previously described cohort of patients recovering from hand paresis after first-ever ischaemic stroke in SM1 using tensor-based morphometry (TBM) of serially acquired, high-resolution T1-weighted images. TBM is based on the analysis of high-dimensional deformation fields necessary to match sets of images and has been repeatedly used to detect subtle GMV changes in longitudinal neurological studies (Agosta et al. 2009; Brambati et al. 2007; Farbota et al. 2012; Kipps et al. 2005). We specifically focused our analysis on the relationship between GMV change and recovery from hand paresis, as the latter represents a very common and clinically highly relevant lateralized impairment. We chose deliberately to investigate a time frame outside the acute phase to avoid the cofounding effects of local oedema and inflammation. We hypothesized that GMV changes in both perilesional and remote subcortical grey matter would relate to recovery of dexterous hand function at the subacute stage after ischaemic stroke.

## Patients and methods

We prospectively recruited patients at two comprehensive stroke centres (Department of Neurology, University Hospital Bern and Kantonsspital St. Gallen, Switzerland) from 01 January 2008 through 31 July 2010. The study received approval from ethical committees at both research centres, and all participants gave written informed consent before enrolment according to the Declaration of Helsinki. Inclusion criteria were (1) first-ever stroke, (2) clinically significant contralesional hand plegia or paresis as a main symptom, and (3) involvement of the pre- and/or post-central gyrus as indicated by hyperintensity on diffusion-weighted (DWI) and fluid attenuated inversion recovery (FLAIR) images at admission. Excluded were patients with (1) aphasia or cognitive deficits severe enough to preclude understanding the study purposes and instructions, (2) prior cerebrovascular events, (3) occlusion of the carotid arteries on MR angiography, (4) purely subcortical stroke, and (5) other medical conditions interfering with task performance. All patients received intensive inpatient neurorehabilitation appropriate to their functional impairment and clinical needs during the first 3 months. No targeted intervention was regularly provided afterwards. Out of 36 recruited patients, seven had to be excluded (three withdrew consent, two were too frail for repeated testing, one was shown to have no cortical stroke after enrolment, and one was lost to follow-up). For the present analysis, one additional patient (one woman) had to be excluded because of MR motion artefacts. The final sample consisted of 28 patients. As controls for behavioural norm values, we used a group of 22 healthy seniors [11 male, mean age 67.6 years (range 45–79)], matched for age [unpaired two-tailed *t* test: *t*(48) = 3.2, *p* < .19]. Data on these patients and controls have been published previously (Abela et al. 2012).

### Behavioural data

#### Behavioural data acquisition

We tracked post-stroke recovery over ten visits (baseline within the first week after stroke plus nine monthly examinations) with standardized tests of clinical outcome, motor and somatosensory function. All examinations were performed by the same investigator (EA) at both sites. Clinical assessment included the National Institute of Health Stroke Scale (NIHSS) (Brott et al. 1989) and modified Rankin Scale (mRS) (Bonita and Beaglehole 1988). Motor functions of each hand were measured with hand dynamometry (HD) (Mathiowetz et al. 1984) and the Jebsen–Taylor test of hand function, a standardized quantitative assessment that consists of seven timed subtests that simulate everyday activities (Jebsen et al. 1969). We used a modified version (mJTT) that includes only the five subtests with highest stability and test–retest reliability (Stern 1992): (1) turning five index cards, (2) picking six small common objects (two paper clips, two bottle caps, and two coins) and dropping them into an empty can, PSO, (3) stacking four checkers on a board, (4) lifting and moving empty cans, and (5) lifting and moving heavy cans. Whereas HD can be achieved with a whole-hand power grip, mJTT subtests require predominantly precision grips that are characterized by varying patterns of thumb opposition against one or two fingers (Castiello 2005). Both types of grips require physiologically different aspects of motor control and engage different sensorimotor and fronto-parietal networks (Binkofski and Buccino 2004). For somatosensory assessment, we recorded pressure perception thresholds with graded monofilaments. The mJTT was recorded at each visit (ten measurements), all other test at baseline, 3 months, and 9 months (three measurements). For detailed examination methods, see Supplementary Materials.

#### Response feature analysis of motor recovery

In order to accurately describe motor recovery and overcome common problems of longitudinal data (serial correlations, time-dependent interindividual variability), we adopted a variant of response feature analysis (RFA) that refines our previous efforts in modelling hand function recovery (Matthews et al. 1990; Abela et al. 2012). In sum, RFA consists of deriving a single number that best summarizes a salient characteristic of individual time-dependent change (“response feature”) and using this new measure to compare groups or calculate correlations with covariates (Matthews et al. 1990). To this end, we first used a model-based classification of the patient cohort into recovery subgroups, using linear and exponential functions to fit each patient’s recovery trajectory (steps 1–3 below). As a new addition, we performed a principal component analysis (PCA) on the recovery data (step 4). This step assigns a single number to each patient that indicates his position within a continuum of recovery. Thus, RFA leads to two complementary, categorical and continuous, descriptions of individual recovery.

In detail, we proceeded as follows: (1) First, each patient’s mJTT data were transformed to *z*-scores using the mean and standard deviation of the healthy control group, such that negative values corresponded to greater impairment. Normal performance was defined as *z* = 0 ± 2.5 units. (2) mJTT subtests were then ranked to identify the task that would capture dexterous recovery best, according to the following criteria: strongest longitudinal effects (*p* < .001), largest within-subjects variability, and highest proportion of patients with poor recovery at 9 months. (3) Each patient’s recovery trajectory (per mJTT subtest) was identified by fitting a set of linear and exponential models to the *z*-scores of the best task, and the best-fitting model was selected using Akaike’s information criterion (Anderson and Burnham 2010). Patients were classified in three recovery subgroups according to their recovery model: fast (linear recovery trajectory), slow (exponential recovery trajectory that converges to *z* > −2.5), and poor recovery (exponential recovery trajectory that converges to *z* ≤ −2.5). (4) A PCA was performed of the longitudinal mJTT data, and the first principal component (PC) was used to calculate single-subject PC scores (or expression coefficients, see Supplementary Materials for details). Conceptually, the first PC corresponds to a global recovery trajectory that explains most of the variance of the longitudinal z-score data of the task selected in step 2 above, and the PC scores represent the projection of each subject’s recovery trajectory onto this first PC. Thus, this step leads to a single number or “response feature” that represents the expression of the global recovery trajectory by one subject.

### Imaging data

#### Imaging data acquisition

High-resolution T1-weighted MR images were obtained by an optimized 3D modified driven equilibrium Fourier transform (MDEFT) sequence at 3 and 9 months after stroke on the same 3T Siemens Magnetom Trio system (Erlangen, Germany) equipped with a 12-channel radio-frequency head coil (Deichmann et al. 2004; Ugurbil et al. 1993). This sequence provides optimized signal-to-noise and contrast-to-noise ratios for grey and white matter and leads to superior tissue segmentation results in voxel-based morphometry studies (Mordasini et al. 2012; Tardif et al. 2009). The acquisition parameters were as follows: 256 × 256 × 176 matrix points with a non-cubic field of view of 256 mm × 256 mm × 176 mm, yielding a nominal isotropic resolution of 1 mm^3^, repetition time TR = 7.92 ms, echo time TE = 2.48 ms, flip angle = 16°, inversion with symmetric timing (inversion time 910 ms), fat saturation, 12 min total acquisition time.

#### Tensor-based morphometry

We performed a whole-brain TBM analysis with Statistical Parametric Mapping 8 (SPM8, version 4667; www.fil.ion.ucl.ac.uk/spm/) for MATLAB (R2009a, MathWorks, Natick, MA, USA). TBM is an analysis technique that quantifies 3D, voxel-wise patterns of volumetric change by calculating the gradient of a deformation field necessary to warp one MR image to another. Following the original publication (Kipps et al. 2005), we proceeded as follows: (1) we first identified the ischaemic tissue on three- and nine-month T1 images by visual inspection and manually drawn binary lesion masks with MRIcron (www.mccauslandcenter.sc.edu/mricro/mricron/) as previously described (Abela et al. 2012). Lesion volumes were calculated by summing all in-mask voxels. (2) T1 images from both time points were rigidly registered by maximizing the normalized mutual information of the joint intensity histograms (Maes et al. 1997) and corrected for intra-subject bias differences using the VBM8 toolbox (http://dbm.neuro.uni-jena.de/vbm/). Registration parameters were applied to the lesion masks. Images with left-sided lesions and corresponding masks were flipped to the right. (3) A high-dimensional deformation field was calculated that described the warps necessary to match the early to the late image point-by-point by minimizing the mean squared difference between the three- and nine-month images (SPM8, high-dimensional warping algorithm, eight iterations). The regularization parameter that defines the trade-off between the mean squared image difference and the smoothness of the deformations was set to four. The amount of regional volume change (increase or decrease) was quantified by calculating the Jacobian determinant (JD) of the deformation field at each voxel. The JD is a feature of the deformation field that encodes volume change, and voxel values of the JD in our case indicate the amount of local volume increase or decrease relative to the first image. (4) Three-month images were segmented into partial volume maps of grey matter (GM), white matter, and cerebro-spinal fluid using SPM8’s unified segmentation algorithm with cost-function masking to avoid image distortions and minimize segmentation errors (Andersen et al. 2010; Ashburner and Friston 2005; Brett et al. 2001). This procedure rests on excluding lesional and perilesional voxels from the segmentation/normalization algorithm using binary masks and compares favourably against newer segmentation algorithms and importantly does not lead to lesion shrinkage during normalization (Andersen et al. 2010; Ripolles et al. 2012). (5) To arrive at a tissue-specific map of GMV change, JD maps were then multiplied voxel by voxel with a GM segmentation of the image using the following formula: (JD value − 1)*GM. Units of GMV change maps are *n* mm^3^ of GMV at 3 months per 1 mm^3^ of GMV at 9 months. (6) These maps were warped into the stereotaxic Montreal Neurological Institute (MNI) space using normalization parameters derived from step (4). Normalized GMV change maps were finally smoothed with a 12-mm isotropic Gaussian kernel, motivated by previous studies that show a reduction of false positives for this kernel size in voxel-based morphometry studies (Salmond et al. 2002).

### Statistical analysis

#### Mass-univariate analysis

Imaging data were analysed using voxel-wise mass-univariate statistics within the framework of the general linear model in SPM8 (Ashburner and Friston 2000). We performed two analyses: first, to test whether there were any significant GMV changes across time for the whole patient cohort and second, to test whether there were significant linear correlations between GMV and motor recovery scores derived from RFA, over and above differences in clinical variables.

The first analysis was implemented as a one-sample *t* test with the smoothed GMV change maps as a dependent variable, and age, gender, lesion size, average GMV change, and time difference between acquisition dates as nuisance covariates. The second analysis was implemented as a multiple regression model, with the smoothed GMV change maps as a dependent variable, the RFA-derived recovery measure as an independent variable, and age, gender, lesion size, average GMV change, difference between acquisition dates, and differences in power grip (HD) and NIHSS score between examinations as nuisance covariates. To protect against partial volume effects (PVE) and reduce the amount of voxel-wise tests, models were estimated within a grey matter analysis mask that excluded the lesion core (see Supplementary Figure S1 and below for quality control measures). To generate this mask, grey matter segmentations and lesion images were averaged, and binary masks of both images types were created by an iterative, operator-independent algorithm that maximizes the correlation between mask and average (Ridgway et al. 2009).

For both analyses, the threshold for significance was set to *p* < .05, family-wise error (FWE) corrected for multiple comparisons with threshold-free cluster enhancement (TFCE) (implemented for SPM8 by Gaser et al. http://dbm.neuro.uni-jena.de/tfce/) (Smith and Nichols 2009). The TFCE algorithm transforms an unthresholded statistical parametric map (here an SPM{t}) such that the intensity of cluster-like structures within that image is (nonlinearly) enhanced compared to background noise. This is achieved by assigning each voxel of the SPM{t} a new value (or score) that corresponds to the amount of “local spatial support” for that voxel. Spatial support corresponds to the sum of all voxels with less significant t-values. TFCE optimises the detection of both very focal signals with high amplitude, as well as low-amplitude, spatially extended signals. Critical thresholds and *p* values for this new, spatially enhanced SPM{t} were then determined via permutation-based testing, i.e. by deriving an empirical TFCE score distribution from the data using 10,000 permutations in our case. Effects that did not meet the significance criterion are reported as exploratory.

#### Region of interest analysis

To test for regionally specific effects across subgroups, we performed a post hoc region of interest (ROI) analysis with independent atlas-derived ROIs, using the Jülich cytoarchitectonic probabilistic atlas (SPM Anatomy toolbox, Version 1.8, made available through the Human Brain Mapping division at the Forschungszentrum Jülich at, http://www.fz-juelich.de/inm/inm-1/DE/Forschung/_docs/SPMAnatomyToolbox/SPMAnatomyToolbox_node.html). We first identified the cytoarchitectonic localization of each statistically significant [*p* (FWE) < .05] GMV cluster detected in the mass-univariate analyses by calculating its overlap with the maximum probability maps (MPMs) of each cytoarchitectonic area (Eickhoff et al. 2005, 2006). Only overlaps >10 % are reported. For each significant cluster, we then calculated its “central tendency” with respect to the MPMs, i.e. whether it was located more peripherally or more centrally on the underlying cytoarchitectonic area. Central tendency is quantified as a ratio of probabilities, i.e. the mean probability of an area within the overlap with the GMV cluster against the mean probability of that area across the whole brain. Values >1 indicate more central, values <1 more peripheral location. Finally, all MPMs that overlapped with significant clusters were used to generate binary ROI masks, from which average values of confound-adjusted GMV change were extracted for each subject using the Marsbar toolbox (http://marsbar.sourceforge.net/). The complete MPM rather than only the overlap was used to avoid overfitting the data. The extracted GMV values were used for between-group comparisons.

All variables (behavioural and ROI data) were tested for normality using the Kolmogorov–Smirnoff test. Variable transformation or nonparametric statistics were used if appropriate. Coordinates of clusters and peaks are given in MNI space.

### Post hoc quality control

The presence of lesion tissue can affect several steps of image preprocessing algorithms, i.e. longitudinal coregistration, spatial normalization, and particularly tissue segmentation. One problem of the latter is that due to the finite resolution of the anatomical images, any given voxel will contain a mixture of tissue (so-called PVE), and this phenomenon is likely to aggravate segmentation errors, especially in perilesional tissue, where normal tissue classes might be mixed with lesioned tissue. To address these issues, we included the following three quality control measures: first, to estimate the effects of lesioned tissue on coregistration and normalization, we calculated a standard deviation map of the whole cohort and inspected it for perilesional increases of spatial variation (Abela et al. 2012). Second, we estimated the relative PVE error within each significant GMV cluster using a robust algorithm that simultaneously detects voxels of unique and unambiguous tissue classes as well as voxels that contain more than one tissue type and is not based on tissue priors (Tohka et al. 2004). To estimate relative PVE errors, we computed the ratio between the number of GM voxels found with this PVE algorithm against the number of GM voxels found by the SPM8 segmentation described above, for every time point and significant GMV cluster, in single-subject space. For this comparison, all voxels >0.8 were defined as GM tissue as obtained by SPM8. Thirdly, we calculated the central tendency (see above) for each GMV cluster against the lesion probability map of each subgroup. This allowed us to compare the relative topography of necrotic tissue and GMV effects, as a complement to the PVE error calculations.

## Results

### Clinical findings

Demographic and clinical characteristics at baseline are summarized in Table 1. There were more men than women in our cohort, and slightly more left- than right-sided strokes. As determined by the NIHSS score, patients were mildly to moderately affected, and their disability scores ranged from no significant to moderately severe disability. Between left- and right-side stroke patients, there were no statistically significant differences in age (unpaired two-sided *t* test: *t*(26) = 0.93, *p* < .36), NIHSS (Mann–Whitney *U* test: *U* = 89, *p* < .74) or mRS (*U* = 94, *p* < .92). Coincidentally, all four women had right-sided strokes. On clinical examination, none of the patients had relevant spasticity of the affected upper limb.

**Table 1:** Baseline demographic and clinical characteristics

Id	Age (years)	Sex	Stroke side	Lesion location	Volume (cc)	NIHSS	mRS	MMSE
p01	77	m	L	SM1	2.7	4	2	29
p02	50	m	R	SM1, PMC	14.8	7	4	30
p03	78	m	R	SM1	2.8	5	3	27
p05	80	m	L	SM1	2.5	2	4	27
p06	53	f	R	SM1, PMC, PPC, SII	22.0	6	2	28
p07	78	f	R	SM1, PMC, PPC	17.7	4	3	30
p09	70	f	R	SM1, PMC, PPC, SII	75.5	3	2	26
p11	41	f	R	SM1, SII	5.9	3	2	27
p12	54	m	R	SM1	8.2	4	1	30
p15	54	m	L	SM1, PPC	22.1	6	3	30
p16	73	m	R	SM1	2.7	4	2	30
p17	58	m	L	SM1	18.3	4	3	30
p20	70	m	L	SM1, PPC, SII	67.9	6	3	30
p24	74	m	R	SM1, PPC	24.9	4	1	30
p25	49	m	R	SM1, PPC, SII	70.4	3	2	28
p26	44	m	L	SM1	2.1	3	1	30
p30	63	m	L	SM1	9.7	4	2	26
p31	63	m	L	SM1	0.8	5	3	27
p33	75	m	R	SM1	12.1	3	2	30
p35	78	m	L	SM1	0.7	5	2	26
p36	60	m	L	SM1, PMC	3.6	4	3	27
p37	75	m	R	SM1, PPC	63.6	4	3	30
p38	77	m	L	SM1, PPC	3.4	5	2	30
p41	51	m	R	SM1	0.6	2	3	30
p42	64	m	R	SM1	1.9	1	2	30
p43	82	m	L	SM1, PPC	6.7	3	2	28
p44	67	m	R	SM1, PMC, PPC	141.7	11	4	27
p45	53	m	R	SM1, PMC, PPC, SII	78.8	14	4	27
	64.7 (41–82)*	m (24) f (4)	R (16) L (12)	SM1(28), PPC (12) PMC (7), SII (6)	24.4 (0.6–141.7)*	4 (1–14)	2 (1–4)	28 (26–30)

*cc* Cubic centimetres, *Id* study identification number, *NIHSS* National Institutes of Health Stroke Scale, *mRS* modified Rankin Scale, *MMSE* Mini-Mental State Examination, *SM1* primary sensorimotor cortex, *SII* secondary somatosensory area, *PMC* premotor cortex, *PPC* posterior parietal cortex, *L* left, *R* right

* Mean (range), otherwise median (range). Lesion location was visually identified on acute diffusion-weighted images

### Motor recovery

Longitudinal clinical and sensorimotor data of the comprehensive evaluation at baseline, 3 months, and 9 months are summarized in Table 2 including NIHSS with detailed upper limb and cognitive subtasks, mRS, all mJTT subtests and global mJTT score, and HD and pressure perception. Of all mJTT subtests, PSO showed by far the highest proportion of patients that scored outside the defined normal performance threshold at 9 months (8 out of 28), the largest differences between baseline and 9 months (mean ± SD 9.1 ± 4.5 s) and the largest within-subjects variance (mean ± SD 40.4 ± 23.2). Furthermore, the other mJTT PC scores were significantly correlated to the PSO PC scores (see Supplementary Table 1) and, thus, redundant, with diminishing classification power in the order shown in Table 2. Of note, this ranking corresponds to the level of precision grip (and thus manual dexterity) each subtest requires. Thus, based on our subtest ranking criteria (see Methods), PSO represents the most suitable indicator of dexterous hand function. Note that PSO necessitates both reaching to an object and grasping with precision grip; thus, PSO impairment could results from dysfunction in either of these actions. However, our data show that mJTT subtests relying predominantly on reaching (lifting light or heavy cans) were only mildly affected and recovered fast, indicating that reaching impairment played only a minor role in these patients.

**Table 2:** Longitudinal clinical and sensorimotor data

	Controls	Patients		
		Baseline	Month 3	Month 9
NIHSS (points)^a^				
Upper limb motor function	n.a.	4 (1–4)	3 (0–4)	2 (0–2)
Upper limb sensory function	n.a.	3 (0–3)	2 (0–2)	1 (0–1)
Cognitive function	n.a.	2 (0–3)	1 (0–2)	0 (0–1)
Sum	n.a.	4 (1–14)	2 (0–12)	1 (0–7)
Modified Rankin Scale (points)^a^				
Sum	n.a.	2 (1–4)	1 (0–3)	1 (0–3)
Modified Jebsen–Taylor test (s)^b^				
Picking small objects	5.7 (4.6–7.5)	17.0 (5.3–76.1)	10.7 (4.0–45.5)	8.5 (4.7–24.1)
	6.1 (4.7–8.3)	7.5 (3.9–15.1)	6.2 (3.9–10.4)	6.0 (1.4–9.8)
Stacking checkers	4.5 (3.4–7.5)	15.8 (6.0–63.1)	8.3 (3.6–24.6)	6.3 (2.5–18.7)
	5.0 (3.5–8.1)	7.2 (6.0–26.0)	5.1 (3.1–12.9)	4.3 (2.3–8.2)
Turning cards	4.5 (5.0–9.2)	11.0 (5.3–44.4)	6.6 (4.1–31.1)	4.4 (2.5–13.2)
	4.8 (3.1–10.7)	6.5 (3.3–15.1)	4.5 (3.8–9.2)	4.3 (2.7–7.3)
Lifting light objects	4.1 (3.0–5.2)	7.4 (5.4–31.7)	5.4 (2.4–17.1)	4.3 (2.6–10.2)
	4.2 (3.2–5.3)	5.0 (2.9–8.9)	3.9 (2.5–7.8)	3.4 (2.5–5.6)
Lifting heavy objects	4.0 (2.6–6.4)	7.8 (5.4–49.9)	5.0 (2.4–14.8)	4.2 (2.1–7.5)
	4.1 (2.9–6.8)	5.1 (2.7–10.4)	3.7 (2.5–6.2)	3.5 (2.5–5.9)
Sum	22.3 (18.9–28.6)	57.9 (23.1–240.6)	35.2 (16.6–130.7)	26.9 (15.2–69.0)
	24.0 (19.1–30.7)	31.6 (18.4–58.0)	23.3 (15.7–43.6)	21.6 (14.6–31.8)
Hand dynamometry (kg)^b^	36.4 (16.0–61.0)	21.4 (0.0–51.0)	32.4 (9.0–59.0)	37.1 (10.0–67.0)
	35.3 (16.0–59.0)	40.9 (16.0–63.0)	43.0 (13.0–63.0)	43.9 (16.0–67.0)
Pressure perception (g/mm^2^)^b^	8.0 (5.2–10.0)	38.6 (7.7–178.0)	19.5 (5.7–178.0)	20.4 (6.8–155.3)
	7.8 (5.8–11.0)	11.3 (5.2–39.8)	10.5 (6.2–20.5)	10.1 (5.0–18.8)

Values are mean (range) except were indicated

*NIHSS* National Institute of Health Stroke Scale (“Cognitive Function” is the sum of aphasia and neglect items), *n.a.* not applicable

^a^Median (range)

^b^Upper row: values for contralesional hand of patients, right hand of controls, lower row: values for ipsilesional hand of patients, left hand of controls

**Fig. 1:**
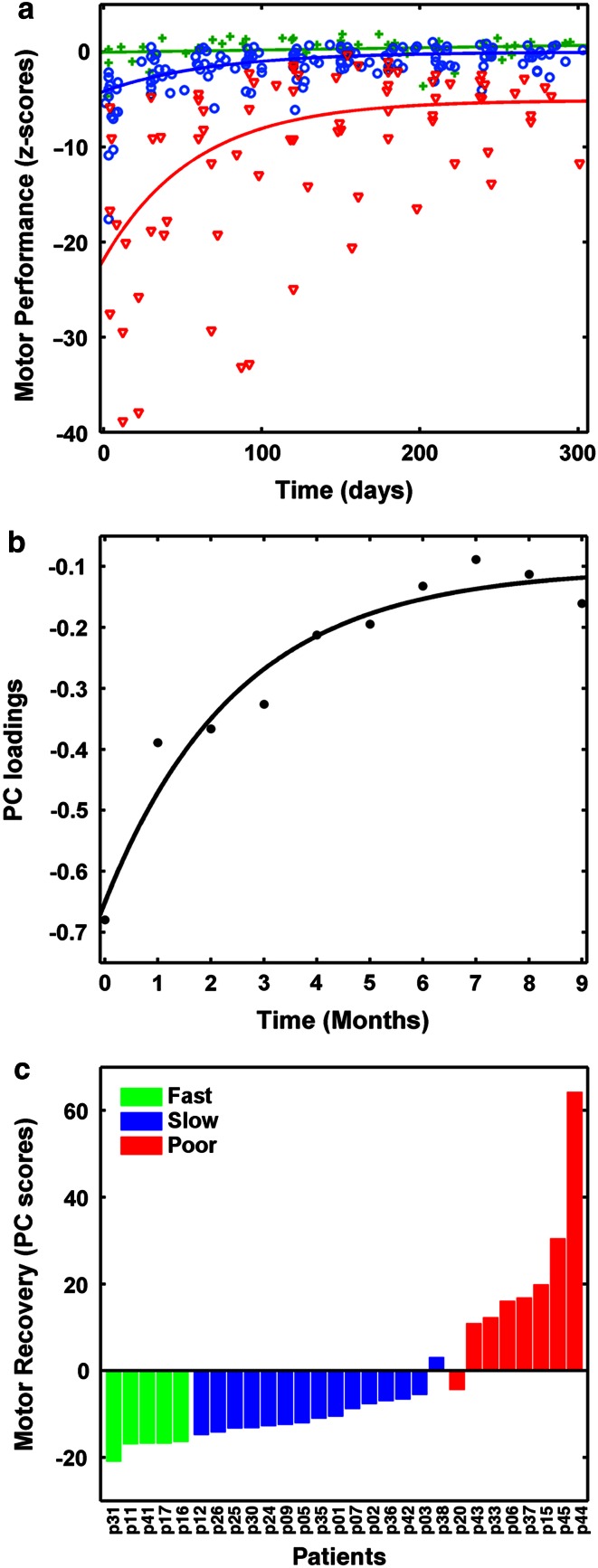
Results of motor recovery analysis. **a** Summarizes the single-subject motor performance values on the “picking small objects” task (*z*-scores against time) and modelled average recovery trajectories of each patient subgroup (green crosses, fast recovery; *blue circles*, slow recovery; *red triangles*, impaired recovery). A *z*-score of *zero* indicates the mean of healthy control performance. **b** Depicts the loadings of the first principal component (PC), and their associated exponential fit. Loadings were calculated from PSO scores measured at each monthly visit (0, baseline; 9, final visit after 9 months). **c** Shows the single-subject PC scores. These values correspond to the projection of each subjects’ recovery trajectory on the first PC. *Lower values* indicate faster recovery, higher values increasing chronic deficit. Recovery subgroups cluster along a continuum of motor recovery, with some degree of overlap between slow and poor recovery subgroups. Patient identification number as in Table 1

Next, using model fits to the PSO data, we identified five patients in the fast recovery subgroup, 15 in the slow recovery subgroup, and eight in poor recovery subgroup. Table 3 summarizes subgroup model formulas and averaged model parameters. The latter were used to draw subgroup recovery trajectories (Fig. 1, Panel A). The first PC from PSO data explained 70 % of variance and showed an exponential time course (Fig. 1, Panel B). Patients scored higher on this PC if they expressed an exponential recovery trajectory with chronic impairment and low if their recovery trajectory was linear (Fig. 1, Panel C). Median PC recovery scores increased significantly across subgroups [medians (range) fast = −16.5 (−16.4 to −21.0), slow = −11.1 (−14.9 to 3.1), poor = 16.5 (−5.9 to 64.3), Kruskal–Wallis test: *H* = 21.43, *p* < .001, post hoc Mann–Whitney *U* tests: fast versus slow: *U* = 0, *p* < .001, fast versus poor, *U* = 0, *p* < .002, slow versus poor *U* = 2, *p* < .001]. Again, there was no significant difference in PC recovery scores between left- and right-sided strokes (*U* = 105, *p* < .68).

**Table 3:** Subgroup recovery models

Subgroup (*n*)	Model formula	Model parameters (95 % CI)		
		Initial deficit *I*	Recovery rate *β*	Chronic deficit *c*
Fast (5)	*m* = *I* + *βt*	−0.9 (0.3, −2.4)	0.005 (0.002,0.007)	–
Slow (15)	*m* = I*exp^(−*βt*)^	−5.9 (−2.8, −10.2)	0.023 (0.011,0.045)	–
Impaired (8)	*m* = I*exp^(−*βt*)^ + *c*	−23.4 (−9.5, −45.6)	0.031 (0.045,0.076)	−5.5 (−8.3, −3.4)

The dependent variable m in each model represents motor performance (in *z*-scores, i.e. units standard deviation of healthy control behaviour), the independent variable t represents time in days (starting from the day of stroke) and c chronic deficit. Consequently, units for *I* and *c* are *z*-scores and units for *β* are day^−1^. A negative score in *I* and *c* indicates lower performance compared to healthy controls (normal performance: *z* = 0 ± 2.5). As per definition, fast and slow recovery subgroups exhibit no chronic deficit

### Lesion data

**Fig. 2:**
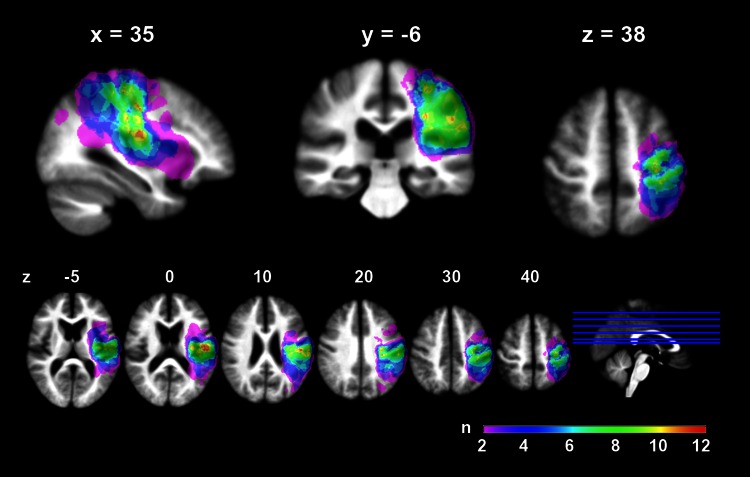
Lesion distribution. A summary lesion map of all individual lesions rendered onto sagittal, coronal, and axial sections (*upper row*) and a series of axial slices (*lower row*) of an average anatomical image from all patients. Colour code indicates number (*n*) of patients with lesion at a given voxel. The colour scale for the lesion overlay map has an upper limit of 12, representing the greatest overlap among the patients in the precentral gyrus (slices *z* = 0–20). All images are in neurological convention (*left side* of the image is left side of the brain). Coordinates are in MNI space (mm)

All patients had lesions in SM1 on visual inspection of the DWI scans (as per our selection criteria). Average lesion volume was 24.9 ± 33.7 cm^3^ (mean ± SD) for the complete stroke cohort. There were no significant differences in lesion volumes between right- and left-sided strokes (Mann–Whitney *U* test: *U* = 133.5, *p* = .082), indicating that flipping images and lesions to one side (see Methods) would not obscure significant hemispheric differences. Lesion volumes were heterogeneous, but there were no lesion volume differences between recovery subgroups [*H* = 5.1, *p* < .08, subgroup medians (range): fast = 5.9 (0.57–22.1) cm^3^, slow = 3.6 (0.7–75.5) cm^3^, poor = 42.84 (2.7–141.7) cm^3^], indicating that any subgroup differences depended on lesion location rather than volume alone. A lesion frequency map (depicting the number of patients with lesion in a given voxel) showed that the lesion core lay in the primary sensorimotor areas and underlying white matter, with variable extension into fronto-parietal and opercular areas (Fig. 2). A detailed analysis of lesion–behaviour relationships in the present cohort has been published before and was beyond the scope of the current analysis (Abela et al. 2012).

### Main effects of GMV change

**Fig. 3:**
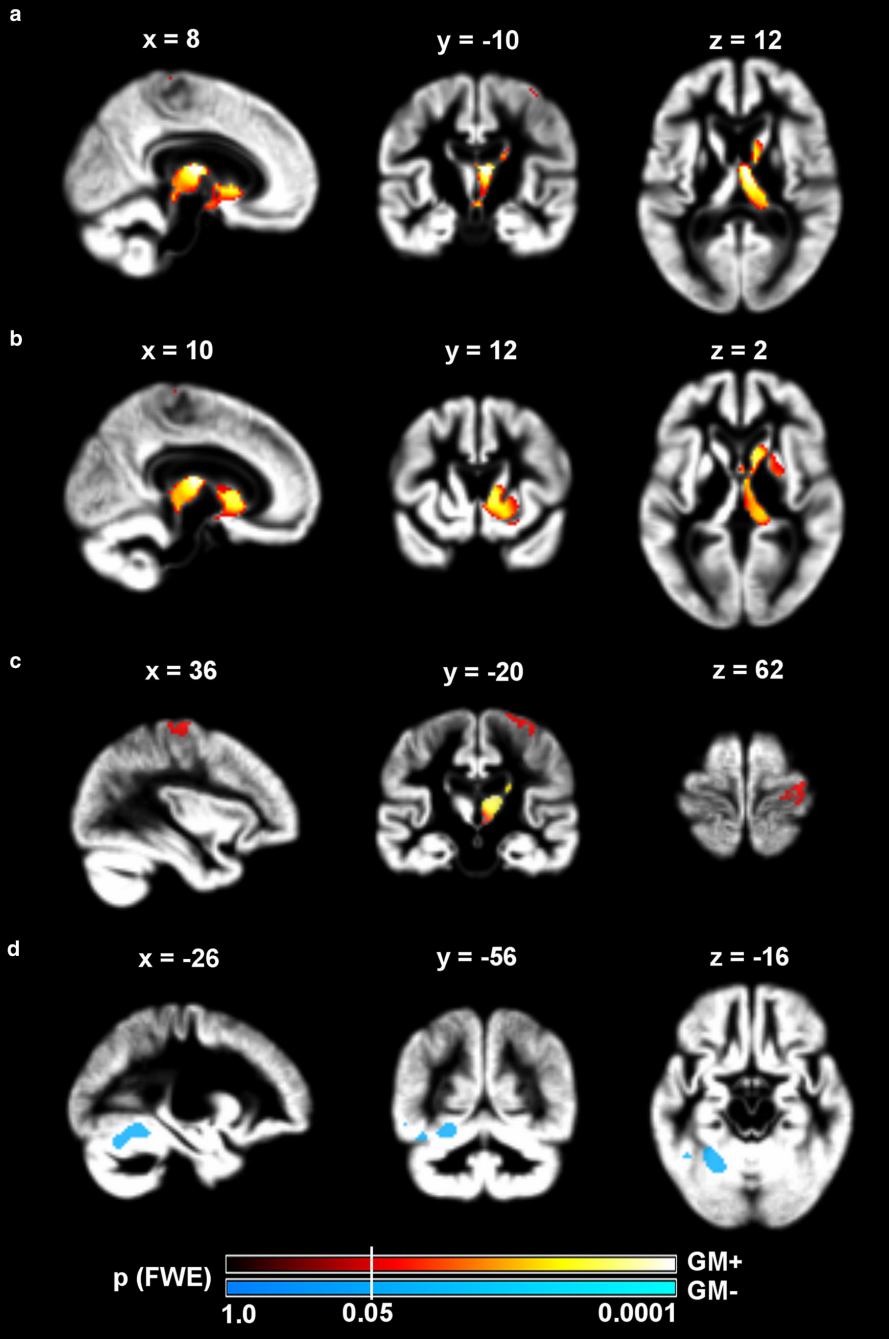
Statistical parametric maps of grey matter volumetric change across all patients. Significant clusters of grey matter volume increase (GM+, hot colours) or decrease (GM−, cool colours) rendered on sagittal, coronal, and axial sections (from *left* to *right*) of an average grey matter segmentation. Sections are chosen to show the maximum effect on the ipsilesional mediodorsal thalamus (**a**), head of the caudate nucleus (**b**), precentral gyrus (**c**), and contralesional cerebellum (**d**). *Colour map* indicates family-wise error (FWE) corrected *p* values at every voxel. Statistical threshold was set at *p*(FWE) < .05 (*white vertical line* across colour bars). All images are in neurological convention (*left side* of the image is left side of the brain). Coordinates are in MNI space (mm)

At the group level, GMV expanded significantly in the ipsilesional precentral gyrus, the mediodorsal thalamus, and head of caudate nucleus. Areas of significant GMV contraction were found in the contralesional anterior cerebellar hemisphere (Fig. 3). Clusters of expansion in the mediodorsal thalamus were assigned using MPMs available in probabilistic atlas; overlap was found with thalamic areas that connect with high probability to prefrontal and temporal cortices, but not with areas that connect to sensorimotor cortices (Behrens et al. 2003). The (perilesional) cluster on the precentral gyrus was found to overlap mostly with probabilistic premotor Area 6 on its dorsolateral aspect (Geyer 2004) and to a lesser extent with the primary motor Area 4a (Geyer et al. 1996) (Table 4).

**Table 4:** Cluster coordinates and statistics for longitudinal grey matter volumetric change

Anatomical area	Cytoarchitectonic area (%)^a^	MNI peak coordinates (mm)			Extent (voxels)	Central tendency	TFCE score	*p* value (FWE)
		*x*	*y*	*z*				
Grey matter expansion: subcortical cluster								
Mediodorsal thalamus	Th-temporal (16.6)	8	−12	10	1,847	1.75	3,399	.0001
	Th-prefrontal (12.2)					1.20		
Caudate nucleus	Caudate head (n. a.)	10	12	2		n.a.	2,420	.0004
Grey matter expansion: cortical cluster								
Precentral gyrus	Area 6 (85.3)	36	−20	62	182	2.22	951	.0293
Precentral gyrus	Area 4a (19.6)	5	−28	75		2.41	890	.0375
Grey matter contraction: cerebellar cluster								
Cerebellum	Lobulus VI (23.8) Lobulus VIIa (11.2)	−26	−56	−16	497	1.44	927	.0307

*FWE* Family-wise error corrected, *n.a.* not assigned in histological atlas, *MNI* Montreal Neurological Institute, *TFCE* Threshold-free cluster enhancement, *Th-temporal/prefrontal* Thalamus with preferential connections to the temporal/prefrontal cortex

^a^Percentage overlap of cluster with cytoarchitectonic probabilistic area (only overlaps >10 % are reported)

**Fig. 4:**
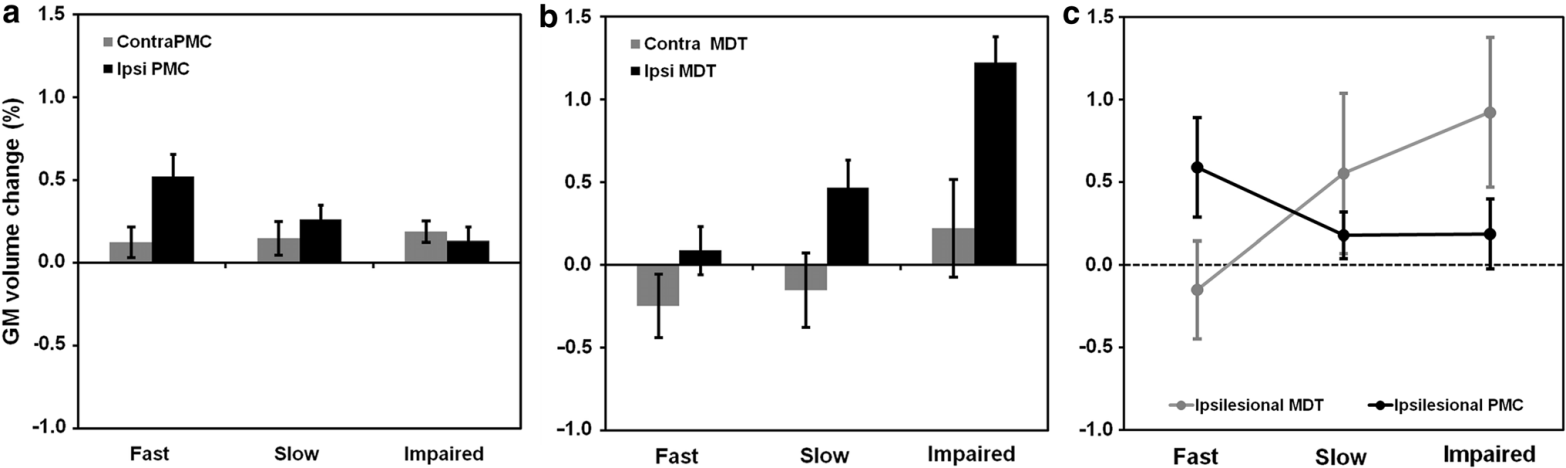
Effect sizes of grey matter volumetric change across subgroups. Panel **a** and **b** show the average grey matter volume changes (% of total grey matter volume) in PMC and MDT. Panel **c** shows the effects of subgroup x locus interaction in the ipsilesional hemisphere. Cortical effects (premotor cortex, PMC) are more pronounced in fast recovered patients, whereas subcortical effects (MDT) are more pronounced in poorly recovered patients. *Error bars* represent 95 % confidence intervals

Confound-adjusted ROI values of GMV increase in the mediodorsal thalamus were significantly different across subgroups [*H* = 8.55, *p* < .014, medians (range): fast = −0.001 (−0.007 to 0.009), slow = 0.004 (−0.003 to 0.029), poor = 0.008 (0.069 to 0.179)]. Post hoc tests revealed that the poor recovery group had a higher thalamic volume expansion compared to the fast group (*U* = 1.5, *p* < .003) and only a trend-level difference to the slow group (*U* = 3.5, *p* < .07) (Fig. 4, Panel B). Conversely, ipsilesional premotor expansion was higher in the fast compared to the poor group (*U* = 15.0, *p* < .05) (Fig. 4, panel A), suggesting an interaction between subgroup membership and locus of GMV change. To test this hypothesis, we performed further analyses on ROI data, extracted as described above. Since GMV data were significantly heteroscedastic [Levene test: *F*(5,49) = 3.74, *p* < .006], we first applied a transformation consisting of adding the lowest negative value (−0.006) to each data point (i.e. shifting the distribution to positive values only) and taking the square root of each new value, leading to normally distributed (Kolmogorov–Smirnov test: *D* = .72, *p* < .68) and homoscedastic data [Levene test: *F*(5,50) = 2.171, *p* < .07]. We then performed a 3 × 2 factorial analysis of variance (ANOVA) with between-subject factor ‘recovery group’ (three levels ‘fast/slow/poor’) and within-subject factor ‘site of effect’ (two levels ‘perilesional/subcortical’). This model yielded a significant group × site interaction [*F*(2, 50) = 4.05, *p* < .02, *η* ^2^ = .159, partial *η* ^2^ = .139], indicating that perilesional cortices underwent significantly more GMV expansion in the fast recovery group, whereas the mediodorsal thalamus grew comparatively more in the poor recovery group (Fig. 4, panel C). Put differently, the ratio of (non-transformed) thalamic to premotor GMV expansion was significantly (*p* < .05) higher in the poor group compared to the two others (see Supplementary Figure S2).

Moreover, the poor recovery group showed significant positive correlations between thalamic GMV expansion and the motor recovery score derived from RFA (Spearman rank correlation: *ρ* = .51, *p* < .005) that was not found in any of the other two groups (both *p* > .5). ROI-based analyses thus show that less successfully recovering patients showed increased GMV expansion in subcortical (striato–thalamic) rather than cortical structures. There were no significant subgroup ROI differences in the contralesional cerebellum (*H* = 1.5, *p* < .47). These effects were thus not explored any further.

### Correlations between GMV change and motor recovery

**Fig. 5:**
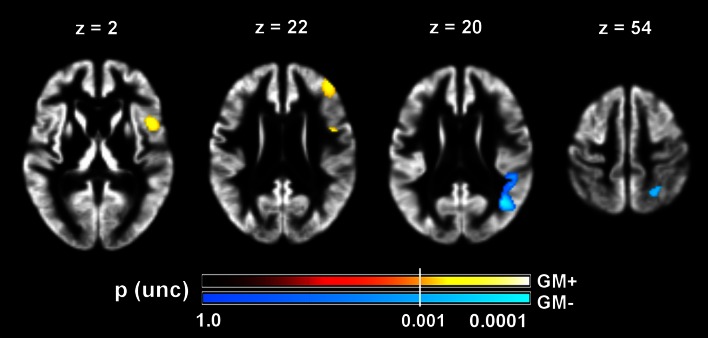
Statistical parametric maps of voxel-wise correlations between grey matter volumetric change and motor recovery. Motor recovery is linearly correlated with grey matter volume increase in the inferior frontal and dorsolateral prefrontal cortex (GM+, hot colours, left axial slices) and grey matter volume decrease in the inferior and superior parietal cortex (GM−, cool colours, right axial slices). Colour map indicates uncorrected (unc) *p* values at every voxel. Statistical threshold was set at *p*(unc) < .001 (*white vertical line* across colour bars). All images are in neurological convention (*left side* of the image is left side of the brain). Coordinates are in MNI space (mm)

We further investigated possible direct voxel-wise correlations between the motor recovery score and GMV change, controlling for age, lesion volume, and differences in HD and NIHSS between 3 and 9 months. Results of this analysis did not survive FWE correction with TFCE and are presented at uncorrected voxel-wise thresholds (*p* < .001) (Table 5; Fig. 5).

**Table 5:** Cluster coordinates and statistics for voxel-wise correlations between longitudinal grey matter volumetric change and motor recovery

Anatomical area	Cytoarchitectonic area (% overlap)^a^	MNI peak coordinates (mm)			Extent (voxels)	Central tendency	TFCE score	*p* value (unc)
		*x*	*y*	*z*				
Ipsilesional positive correlation								
Frontal operculum	Area 44 (14.2)	50	10	2	326	0.84	602	.001
Middle frontal gyrus	BA 46 (n.a.)	41	39	22	186	n.a.	575	.001
Ipsilesional negative correlation								
Angular gyrus	IPC(PGp) (25.0)	40	−64	20	565	1.80	1,169	.001
	IPC(PGa) (16.9)					0.93		
Superior parietal lobule	SPL(7A) (55.8)	26	−60	54	141	1.16	448	.001
	SPL(7PC) (19.2)					1.39		
	hIP3 (11.4)					1.38		

*BA* Brodmann area, *IPC* inferior parietal cortex, *unc* uncorrected, *n.a.* not assigned in histological atlas, *MNI* Montreal Neurological Institute, *TFCE* threshold-free cluster enhancement

^a^Percentage overlap of cluster with cytoarchitectonic probabilistic area (only overlaps >10 % are reported)

GMV change correlated *positively* with the motor recovery score in areas corresponding to the dorsolateral prefrontal cortex (dlPFC) and inferior frontal cortex, indicating that worse recovery correlated with GMV increase in these regions. The peak of dlPFC cluster lay on the medial frontal gyrus, reaching into the superior frontal sulcus, and probably corresponding to Brodmann Area 46 according to online coordinate-based atlases and the current literature,^1^ ^2^ (Cieslik et al. 2012). The peak of the inferior frontal cluster lay on the pars opercularis of the inferior frontal gyrus and could be assigned to the probabilistic cytoarchitectonic Area 44 (Keller et al. 2009). This area is at the core of Broca’s region, which is in turn divided into multiple cytoarchitectonic and functional subunits (Amunts et al. 2010). A recent meta-analysis using functional connectivity data has found five distinct subunits, whose maps have been made publicly available (Clos et al. 2013). Using these maps, we found an overlap of 16.5 % between the inferior frontal cluster of GMV increase and one posterior inferior functional subunit associated with action imitation. No overlap was found with language-related subunits.

GMV change in two clusters in the superior and inferior parietal lobule (SPL and IPL, respectively) correlated *negatively* with the motor recovery score, thus indicating that more pronounced GMV decrease (atrophy) in these regions was associated with less favourable recovery. In terms of associated cytoarchitectonic areas, the inferior cluster overlapped with anterior and posterior portions of the inferior parietal cortex [IPC(PGa) and IPC(PGp), respectively] on the angular gyrus (Caspers et al. 2008). The superior cluster lay at an intersection with two areas located superiorly and medially on the SPL (7A, 7PC) and overlapped a posterior region of the intraparietal sulcus (hIP3) (Scheperjans et al. 2008).

## Results of quality control analyses

A standard deviation map indicated no perilesional increase of spatial variation due to the presence of the lesion (Supplemental Figure S3). Across all subgroups, relative PVE errors within the cortical (perilesional) cluster were on average 0.14 ± 0.37 (mean ± SD) for T1 images acquired at 3 months and 0.18 ± 0.48 for T1 images acquired at 9 months after exclusion of three outliers (at 3 months: patient p43, poor recovery subgroup; at 9 months: patient p37, poor recovery, and patient p41, fast recovery subgroup). Relative PVE errors for the subcortical cluster were 0.27 ± 0.02 and 0.27 ± 0.03 for first and second acquisition time points, respectively (no outliers). The ratio of subcortical versus cortical PVE was not significantly different between time points and subgroups, indicating that differential segmentation errors could not account for the subgroup x cluster location interaction seen in Fig. 4 (for complete PVE statistics, see Supplemental Figure S4 and Supplemental Table 2). Exclusion of outlier subjects did not qualitatively alter results of voxel-wise statistics. Central tendency of the cortical cluster versus each subgroup lesion probability map was low (central tendency for each subgroup: fast = 0.07, slow = 0.05, and poor = 0.5), indicating very peripheral location of the GMV effect with respect to the necrotic lesion tissue, especially in the fast and slow subgroups (Supplemental Figure S5).

## Discussion

In this study, we have identified perilesional and remote cortico-subcortical changes in grey matter morphology that occur during the subacute phase after ischaemic stroke in SM1 and are related to subject-specific recovery trajectories of dexterous hand function. Our analysis of behavioural data used both model-based classification and multivariate analysis to quantify motor recovery during performance of a simple mJTT subtest that requires precision grip and visuomotor coordination. Motor recovery trajectories could be empirically classified into three subgroups, which were associated with distinct morphological correlates that characterize recovery dynamics. Specifically, we found that the neuroanatomical sites of significant GMV change dissociate, such that GMV expansion of the peri-infarct PMC is predominantly seen in patients that recover normal motor hand skill quickly, whereas GMV expansion in striato-thalamic regions is the hallmark of those patients who recover more slowly and remain chronically impaired. Moreover, we found indications that motor recovery dynamics, as indexed by our measure derived from RFA, might be linearly correlated with GMV increase of the ipsilesional dlPFC and inferior frontal cortex, as well as atrophy of clusters in the superior parietal lobule (SPL) and inferior parietal cortex (IPC). It is important to stress that these voxel-wise correlations, in contrast to the whole-group *t* test, did not meet the formal criteria for statistical significance and must be viewed as exploratory. However, there is ample evidence from the literature that the indentified areas are neurobiologically plausible, as discussed below. Note also that effect sizes for both GMV increase and decrease were small (±0.5–1.5 % change), but this is within the same order of magnitude seen in previous studies using similar methods to detect GMV change after subcortical stroke (Gauthier et al. 2008). All analyses were corrected for lesion size and can thus not be explained by infarct volume.

In sum, our results represent a model of interaction between specific sites of neuronal reorganization and degree of disturbed motor recovery, with fast, slow, and impaired functional restitution as dependent variables in the recovery model. The method applied here has been successfully used in previous longitudinal morphological studies, yielding pathophysiologically plausible results in a wide variety of neurological conditions and experimental settings (e.g. Agosta et al. 2009; Brambati et al. 2009; Ceccarelli et al. 2009; Kipps et al. 2005; Tao et al. 2009; Filippi et al. 2010; Farbota et al. 2012). However, to the best of our knowledge, this is the first study to report such effects in human cortical ischaemic stroke. We now discuss each of the neuroanatomical effects in turn.

### Ipsilesional effects in perilesional primary motor and premotor areas

One of our main results is the GMV increase in peri-infarct portions of dorsolateral Area 6 and medial Area 4a in patients with favourable outcome. Structural reorganization of the perilesional cortex that parallels motor recovery has been described in a wealth of animal studies (Nudo and Friel 1999; Nudo 1997). For instance, studies on non-human primates have shown that new intracortical axons sprout from ipsilesional ventral premotor to the primary somatosensory cortex after isolated motor cortex infarction and that these alterations in cortical wiring pattern are accompanied by extensive topographic reorganization of upper limb representations that parallel behavioural recovery (Eisner-Janowicz et al. 2008; Dancause et al. 2005). Zeiler et al. have recently shown that the ipsilesional medial premotor area in rodents reorganizes to support contralateral forelimb recovery of prehension after a first experimental SM1 stroke and that a second infarction to this region re-induces the initial neurological deficit (Zeiler et al. 2013). Comparable results have been reported using functional methods in patients with subcortical stroke, although a direct comparison must be considered with caution due to the different stroke locations and mechanisms. However, it is interesting to note that, similar to the effect attained by re-injury in the study by Zeiler et al., inhibitory transcranial magnetic stimulation (TMS) of the ipsilesional dorsal PMC slowed the recovered paretic hand during a reaction time task in a group of patients with striato-capsular stroke, indicating a causal relationship between ipsilesional PMC function and recovery of hand function (Fridman et al. 2004). The role of PMC in post-stroke recovery is further supported by functional MRI (fMRI) data. A recent meta-analytic study of fMRI studies across heterogeneous experiments and patient cohorts showed that bilateral PMC activity is a salient common feature of paretic limb movements but that good motor outcome depends on the restoration of activity patterns lateralized to the ipsilesional side (Rehme et al. 2012). Using both fMRI and structural imaging in subcortical stroke patients, Schaechter et al. (2006) have shown that increases of cortical thickness co-localize with activations in the somatosensory cortex during tactile stimulation of the affected hand. Collectively, these data support the interpretation that the GMV increases in the dorsolateral Area 6 and medial Area 4a reported here might indeed indicate adaptive reorganization of spared perilesional motor circuits that is beneficial to behavioural outcome, as it is seen in the group with the fastest recovery trajectories.

### Ipsilesional effects in distant thalamic and fronto-parietal areas

The large subcortical effects seen in persistently impaired patients occur within two distinct structures, the mediodorsal thalamus (MDT) and the head of caudate nucleus. Of note, both are densely interconnected and form part of a cortico-striato-thalamic loop that projects to dlPFC and receives afferents from arcuate premotor area in non-human primates and homologous areas in humans (DeLong et al. 1986; Alexander et al. 1986; Binkofski and Buccino 2004; Petrides and Pandya 2009). The preferential connection of the MDT to the head of the caudate, the dorsolateral prefrontal cortex (dlPFC) and the dorsal anterior cingulate cortex (dACC) have been recently confirmed using tractography (Eckert et al. 2012). Thus, the subcortical GMV expansion occurs in structures that participate both in a prefrontal network commonly associated with cognitive control of action execution (Haber and McFarland 2001) and a limbic network that possibly serves attentional and motivational processes (Ongur and Price 2000). The voxel-wise correlation analysis between GMV change and motor recovery also provided support for involvement of the dorsolateral–prefrontal loop further by delineating the dlPFC as participating node. Of note, a recent meta-analysis of functional connectivity data by Cieslik et al. (2012) has found evidence for two different (anterior–ventral and posterior–dorsal) functional subunits within the dlPFC. Interestingly, the analysis by these authors revealed a similar dichotomy as the one described for the MDT above, namely a functional connection of the anterior–ventral dlPFC with the dACC, possibly subserving attention and inhibition processes, and a functional association of the posterior–dorsal dlPFC with the intraparietal sulcus, associated with action execution and working memory. Furthermore, we found an additional cluster in the posterior part of Boca’s area on the frontal operculum, considered a homologue of the monkey’s ventral premotor area F5 (Binkofski and Buccino 2004; Rizzolatti and Arbib 1998). This cluster overlapped a recently (functionally) defined motor execution subregion of the inferior frontal gyrus and frontal operculum, but none of the more widely known language-related areas (Clos et al. 2013). This opercular subdivision of Boca’s area is thought to support the processing of motor actions that need a high degree of sensorimotor control, specifically precision grip (Ehrsson et al. 2001), but also learning of motor sequences (Seitz and Roland 1992), establishing visuomotor associations (Toni et al. 2001), and imagining and imitating motor actions (Binkofski et al. 2004). It should be noted that this zone is also connected by afferents with the intraparietal sulcus (IPS), which has been shown to be preferentially lesioned in the incompletely recovered patients (Abela et al. 2012). In the correlation analysis, the patients suffered GMV decrease in parts of the SPL and IPC depending on the recovery score that quantifies precision grip impairment. Of note, the IPS, together with SPL, prefrontal, and motor areas, has been shown to be part of functional circuits for grasping and precision grip (Castiello 2005).

In sum, the expansion of MDT and head of caudate nucleus as part of the prefrontal loop has been associated definitely with impaired recovery according to RFA. This finding is supplemented by the observed GMV increase of fronto-parietal cortical nodes in relation to the impaired hand motor skill. These cortical nodes, upstream of the lesioned sensorimotor cortices, are either part of the subcortico-cortical prefrontal loop common to the involved subcortical nodes or interrelated with it. From a functional point of view, these plastic changes in cortical (fronto-parietal) and subcortical (striato-thalamic) grey matter evidence enhanced executive motor drive by cognitive control, especially in those patients that suffer from persistently impaired motor performance. Also, these results reiterate the importance of the integrity of posterior parietal cortices and the multimodal associations they support, for post-stroke hand function recovery (Abela et al. 2012).

### Contralesional effects

All patients exhibited circumscribed GMV involution over the time span of 6 months in the contralesional anterior cerebellum without volumetric differences among recovery subgroups, possibly an effect of morphologically established diaschisis in the cortico-cerebellar loop at late stages of recovery (Nocun et al. 2013; Lin et al. 2009). However, there was no change detected by TBM in the contralateral hemisphere to the ischaemic lesion. This contrasts to findings in a heterogeneous population with subcortical stroke of varying extent examined at different time points with a range between 3 and 18 months (Fan et al. 2012) and a recent study in subcortical patients undergoing constrained induced movement therapy (Gauthier et al. 2008) that found widespread bilateral GMV change. Differences are difficult to resolve at this point, but are likely due to both different lesion location (subcortical vs. cortical stroke) and morphometric methods used.

### Limitations

There are a few limitations to consider. First, although the almost 40 patients in the original strictly selected cohort appeared at the beginning of the study to promise a very satisfactory statistical basis, sample sizes in two recovery subgroups were too small to conduct a voxel-wise ANOVA to substantiate the ROI-based results. Larger cohorts should be followed in future studies. However, the emergence of three subgroups among the 28 patients who completed the study as well as the interaction between subgroup and local reorganization are important results of the study that could not have been anticipated. We thus think that our subgroup classification results are not invalidated by small sample sizes, but accurately characterize the patterns of behavioural recovery present among cortical stroke patients. Second, this study looked specifically at late phases of recovery, and findings cannot be generalized to earlier time points. This limitation could be resolved by acquiring high-resolution MR data within the first 3 months after stroke, when most of behavioural recovery occurs (Fig. 1). On the other hand, the focus on late GMV remodelling after stroke is also a strength of our study, as our results indicate that morphological changes, though small, occur well beyond the time frame in which neurorehabilitation is usually administered and clearly differentiate patients with different motor outcome. This indicates that stroke-induced GMV changes might still be amenable to therapy-dependent modulations even late after stroke. However, the present patient cohort was not selected to determine the effects of neurorehabilitative treatment, which might have influenced GMV change additionally. Future studies could thus combine targeted interventions with measures of grey but also white matter plasticity to resolve the effects of motor experience and white matter tract damage on GMV.

### Conclusions

To conclude, we have shown that based on the RFA of the mJTT, an interaction model shows a significant interrelation between recovery subgroups and GMV change. At its extremes—fast recovery versus persisting impaired recovery of motor hand skill—we found fundamentally different patterns of GMV change, reflecting grey matter plasticity that, however, cannot be assigned to a specific underlying mechanism, e.g. axon sprouting, dendritic branching, and synaptogenesis among others (Zatorre et al. 2012). On the one hand, subjects with fast recovery exhibited perilesional GMV increase which corresponds to a model of local reorganization of sensorimotor representation, finally gaining a level of automatic motor behaviour. On the other hand, subjects with most severely and persisting impaired motor hand skill were distinguished by GM enhancement in a largely distributed network involving nodes of the dorsolateral prefrontal loop and inferior premotor cortex which are known to support attention, motor execution, and processing. This corresponds to a model of a compensatory mechanism using cognitive control. Summarizing, the findings reflect the long-term structural adaptation of cortical and subcortical grey matter in response to the severity of a cortical ischaemic stroke.

## Electronic supplementary material

Below is the link to the electronic supplementary material.

Supplementary material 1 (DOCX 1070 kb)

## Footnotes

1 BrainMap database, http://www.brainmap.org/.

2 Brede database, http://hendrix.imm.dtu.dk/services/jerne/brede/brede.html.
