# Supplementary material for: Grey matter volumetric changes related to recovery from hand paresis after cortical sensorimotor stroke
Source: Brain Struct Funct. 2014 Jun 7;220(5):2533–50. doi: 10.1007/s00429-014-0804-y (PMC4549385; doi:10.1007/s00429-014-0804-y)
Supplement: Supplementary file 1 — Supplementary material 1 (DOCX 1070 kb) [file 429_2014_804_MOESM1_ESM.docx]

**SUPPLEMENTAL MATERIAL**

1. **Supplemental Materials and Methods**
   1. **Behavioural Assessment**

*Hand dynamometry( HD):* A Jamar dynamometer was used to measure maximal grip force (i.e. power grip) over three trials, alternating between hands (Mathiowetz *et al.* , 1984). The dynamometer was held in front of the subject with the elbow flexed at 90° and slightly abducted to avoid contact with the trunk. Subjects were instructed to squeeze the lever of the apparatus as strongly as possible without extending the arm. An investigator supported the dynamometer such that the participant could comfortably exert a power grip without the need to additionally stabilize the dynamometer. Power grip (force) values were recorded in kilograms (higher values indicate better performance).

*Dexterous hand function* was measured for both hands using the modified Jebsen-Taylor Test (mJTT), a standardized quantitative assessment of hand function that consists of five timed subtests intended to simulate everyday actions (Jebsen *et al.* , 1969). The test is scored by adding the seconds needed to complete each subtest; standard performance norms are available from the original publication for both sexes and for different age groups (Jebsen *et al.* , 1969). Several studies have used this test to assess hand function in stroke, and the test is sensitive also to subtle deficits of the ipsilesional hand (which was outside the scope of this study) (Sunderland *et al.* , 1999, Sunderland, 2000, Wetter *et al.* , 2005, Chestnut and Haaland, 2008). mJTT values were recorded in seconds (higher values indicate worse performance). The subtests were applied according to the original instructions, as follows:

(1) Turning Cards. The subject turned 5 index cards (7.5 cm x 12.5 cm) that were positioned at 12.5 cm from the front end of the table and spaced 5 cm apart, beginning with card farthest away on the opposite side of the used hand. After this test, a custom-made wooden board (100 x 30 x 3 cm) was placed on the desk at 12.5cm away from the subject for reference.

(2) Picking Small Objects. Two paper clips, two bottle caps and two coins were positioned on the same side as the tested hand, touching the board. The subject had to pick each object, starting with the one farthest away and put them into a can positioned at the body midline.

(3) Stacking Checkers. Four wooden checkers placed against the front of the board were stacked on top of the board. No order was specified.

(4) Lifting Light Cans. Five empty aluminum cans (~14 x 9 cm) were placed in front of the board 5 cm apart with the open end facing down, the middle can in front of the subject’s midline. Cans were lifted by the subject and placed on the board, starting with the can farthest away on the same side as the tested hand.

(5) Lifting Heavy Cans. The same as subtest 4, but with filled cans (450 g each).

*Somatosensory Assessment:* Pressure perception thresholds (PPT) were measured with Semmes-Weinstein monofilaments (Senselab Aesthesiometer, Somedic AB, Hörby, Sweden) using a simple staircase algorithm to reduce testing time and subject fatigue (Dyck *et al.* , 1993).

- 1. **Modelling of Stroke Recovery**

As in our previous work, we used linear and exponential models to fit the individual time courses of dexterous hand function recovery. We recapitulate our previously published model selection procedures for completeness.

First, model fits were calculated automatically using the algorithms implemented in the Curve Fitting Toolbox (Version 2.0) for MATLAB 7.8.0 (MathWorks, Natick, Massachusetts). Starting values for intercept and constant were derived by simple observation of the scatter plots of each subject’s scores against time. We then used model selection procedures based on Akaike’s Information Criterion (AIC; Akaike 1974; and see a further paper (Anderson and Burnham, 2010) for a comprehensive treatment to select the model that gave the most accurate description of each recovery trajectory. AIC reflects the amount of information that is lost when any model *m*_i_ is used to approximate a process *p* that generated the observed data .The AIC for any *m_i_* is given by the equation:


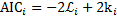
 (1)

where
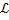
 represents the maximum log-likelihood of the model (given the data) and *k* the number of model parameters. Models with minimal information loss, i.e. smallest AIC value, are preferred. AIC can also be calculated from the residual sum of squares (RSS), the number of observations (*n*) and the number of model parameters (*k*) as:


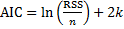
 (2)

When the ratio of *n/k* is small (empirically defined as <40), the use of a small sample correction of AIC is recommended (AICc; Hurvich and Tsai 1989), as defined Burnham and Anderson (2010, p. 66):


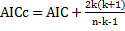
 (3)

Since *n/k* was clearly below 40 in our study (10/4 = 2.5 for the full exponential model) we used Eq. (3), with RSS derived from the curve fitting procedures for our calculations.

To identify the best fitting recovery curve, models were ranked using AICc differences (Δ_i_), which are calculated for *m_i_* as


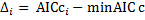
 (4)

where min AICc is the smallest value in the candidate set, i.e. the AICc value of the best approximating model. Based on these differences, the value exp(-1/2Δ_i_) can be used to obtain and estimate of the relative likelihood for each of the candidate models, given the data (Wagenmakers and Farrell, 2004). This quantity is used to calculate Akaike weights (*w_i_*) that represent the evidence favouring one model given the data set and the candidate set of models *m_i_*, i=1...R (Posada and Buckley, 2004, Wagenmakers and Farrell, 2004):


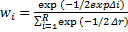
 (5)

A *w_i_* value > 0.9 represents substantial evidence in favor of one model (Burnham and Anderson 2010).

- 1. **Principal Component Analysis**

Each task was analyzed separately with Matlab program, *princomp* [The Mathworks, Inc., Natick, MA], which yielded ten principal component time courses and variances (one per visit), and 28 patient expression coefficients (or “scores”). The Kaiser-Guttmann criterion was used to select salient principal components (Guttmann, 1954). Missing data, arising when patient did not show or could not perform a task, were replaced by means over all patients at the time point of the missing data (n = 10 missed visits out of 280 planned visits).

- 1. **Imaging Data**

**Supplemental Figure S1: Explicit analysis mask**

**
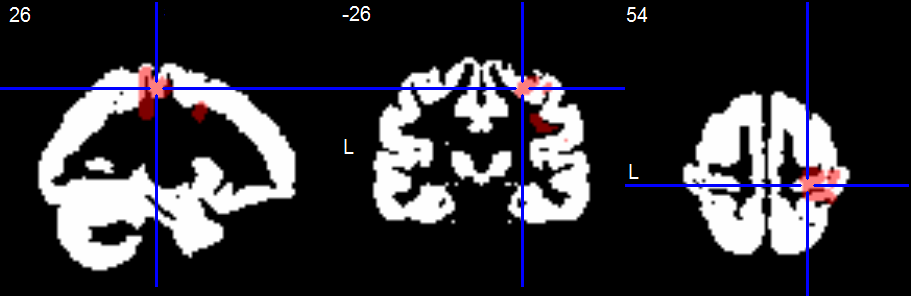
**

This figure shows three sections (sagittal, coronal, axial from left to right) of the binary GM analysis mask (white) with an overlay of the binary lesion mask (red). Note again the lesion core surrounding the central sulcus and some expansion into the white matter. Voxels at the intersection of GM and lesion mask were excluded in the final statistical image analysis. Images are displayed in neurological convention (L, left). Coordinates are in MNI space.

1. **Supplemental Results**

**a) Behavioural Data**

**Supplemental Table 1: Statistics for the first principal components of each modified Jebsen-Taylor subtest**

| **Jebsen-Taylor subtest** | **Var *** | **Eigenvalue** | **r°** | **p-value** |
| --- | --- | --- | --- | --- |
| **Picking Small Objects** | 70% | 458.38 | - | - |
| **Stacking Checkers** | 69% | 254.58 | 0.8 | <.001 |
| **Turning Cards** | 62% | 132.62 | 0.7 | <.001 |
| **Lifting Light Cans** | 72% | 70.80 | 0.7 | <.001 |
| **Lifting Heavy Cans** | 66% | 143.82 | 0.5 | .002 |

***** Variance explained across all patients for each task. ° Pearson's correlation with the first principal component of the picking small objects task. Note the high correlation between subtests.

**b) Imaging Data**

**Supplemental Figure S2: Ratio of subcortical versus cortical grey matter volume change in the three recovery subgroups**


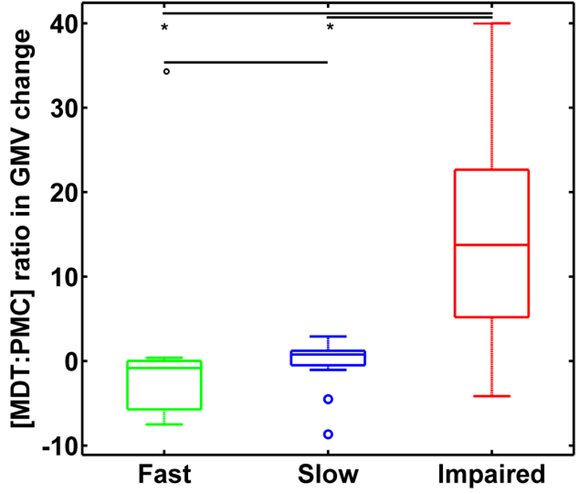


This figure shows boxplots of the ratio of grey matter volume (GMV) change in the mediodorsal thalamus (MDT, ROI "Th-premotor" derived from the cytoarchitectonic probabilisitc atlas) versus change in the premotor cortex (PMC, ROI "Area 6"). Whiskers indicate range between 1st and 4th quartile, solid horizontal bars show the statistical comparisons. There was significantly more subcortical versus cortical GMV change in the impaired, i.e. poor as designated in the paper, recovery subgroup compared to the fast and slow subgroups (*Mann-Withney U-tests, both p<.05). Between the latter, there was a trend-level difference (°Mann-Withney U-tests, p=.07). For definition of subgroups and discussion of results, see main body of manuscript.

**c) Post-Hoc Quality Control**

**Supplemental Figure S3: Quality control of coregistration and normalization- Standard deviation map of normalized T1 images**


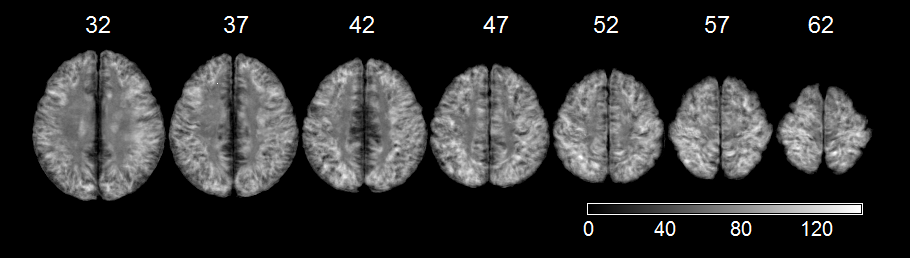


This figure shows axial slices of a voxel-wise standard deviation map of the complete stroke cohort. The lesioned hemisphere is on the right. Images are displayed in neurological convention. Coordinates are in MNI space (z axis).

**Supplemental Figure S4: Quality control of partial volume effects - Graph of relative partial volume estimation errors across subgroups, locations and time points.**

**
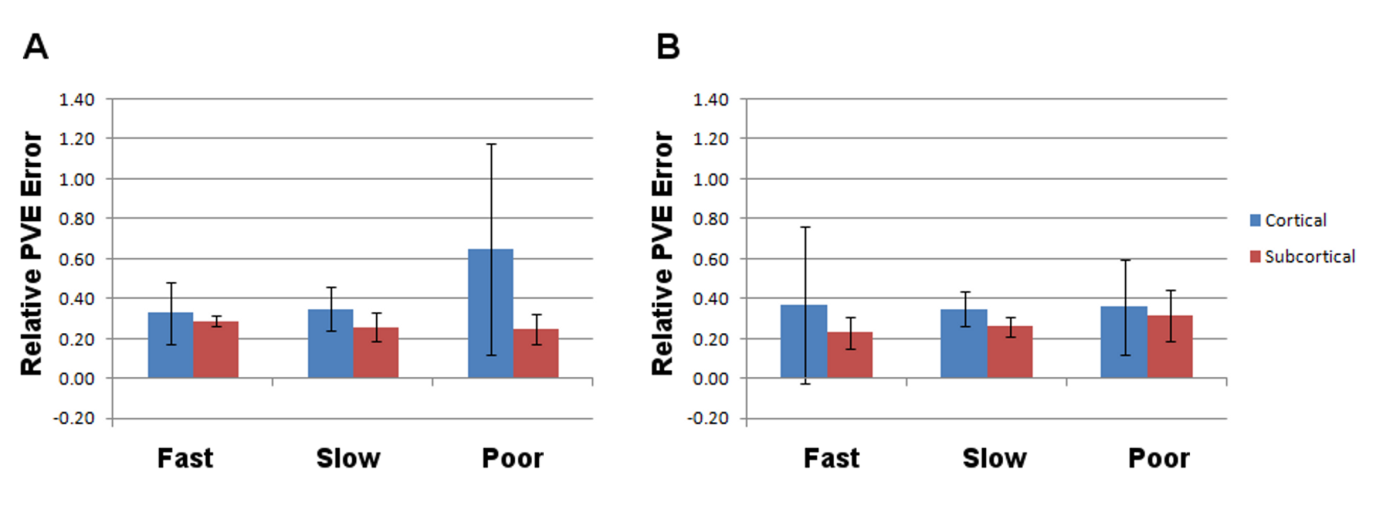
**

This figure shows bar plots of relative partial volume estimation (PVE) errors of grey matter for T1 images at 3 months (panel A) and 9 months (panel B) across recovery subgroups (bars represent mean ± 95% CI).

**Supplemental Table 2: Quality control of partial volume effects - Descriptive and inferential statistics of relative partial volume estimation errors across subgroups, locations and time points.**

| **Subgroup** | **Cortical (oerilesional) rPVE errors*** | | **Subcortical (remote) rPVE errors** | |  | **Ratio of rPVE errors (Subcortical/Cortical)** | | ***W*** | **p** |
| --- | --- | --- | --- | --- | --- | --- | --- | --- | --- |
|  | Month 3 | Month 9 | Month 3 | Month 9 |  | Month 3 | Month 9 |  |  |
| Fast  (n = 5) | 0.33±0.18, 0.11-0.62 | 0.37±0.20, 0.05-1.23^§^ | 0.29±0.03, 0.25-0.32 | 0.23±0.11, 0.08-0.37 |  | 1.22±0.9,  0.51-2.63 | 1.81±2.21, 0.32-5.45 | .14 | .89 |
| Slow  (n = 15) | 0.35±0.20, 0.01-0.73 | 0.35±0.16, 0.11-0.71 | 0.26±0.14, 0.04-0.50 | 0.26±0.05, 0.10-0.44 |  | 2.8±5.1,  0.1-15.8 | 1.01±0.82, 0.17-3.09 | -.9 | .36 |
| Poor  (n = 8) | 0.65±0.76, 0.19-2.52° | 0.36±0.35, 0.04-7.10^#^ | 0.25±0.11, 0.06-0.44 | 0.32±0.09, 0.04-0.61 |  | 0.9±0.9, 0.09-2.54 | 2.70±5.31 0.00-15.65 | 1.1 | .26 |
|  |  |  |  |  | ***K*** | .6 | .1 |  |  |
|  |  |  |  |  | **p** | .74 | .99 |  |  |

* Values are in the following order: mean±SD, range. Maximal values indicate outlier patients: °p43 ^#^ p37, ^§^ p41. Between-time point comparisons: W, Wilcoxon-signed rank-test of relative partial volume estimation (rPVE) error ratios (Month 3 versus 9). Between-group comparisons K, Kruskal-Wallis test of rPVE error ratios (Fast versus Slow versus Poor). p, p-value.

**Supplemental Figure S5: Quality control of relative topography - Overlap of grey matter volume effects with subgroup lesion maps**


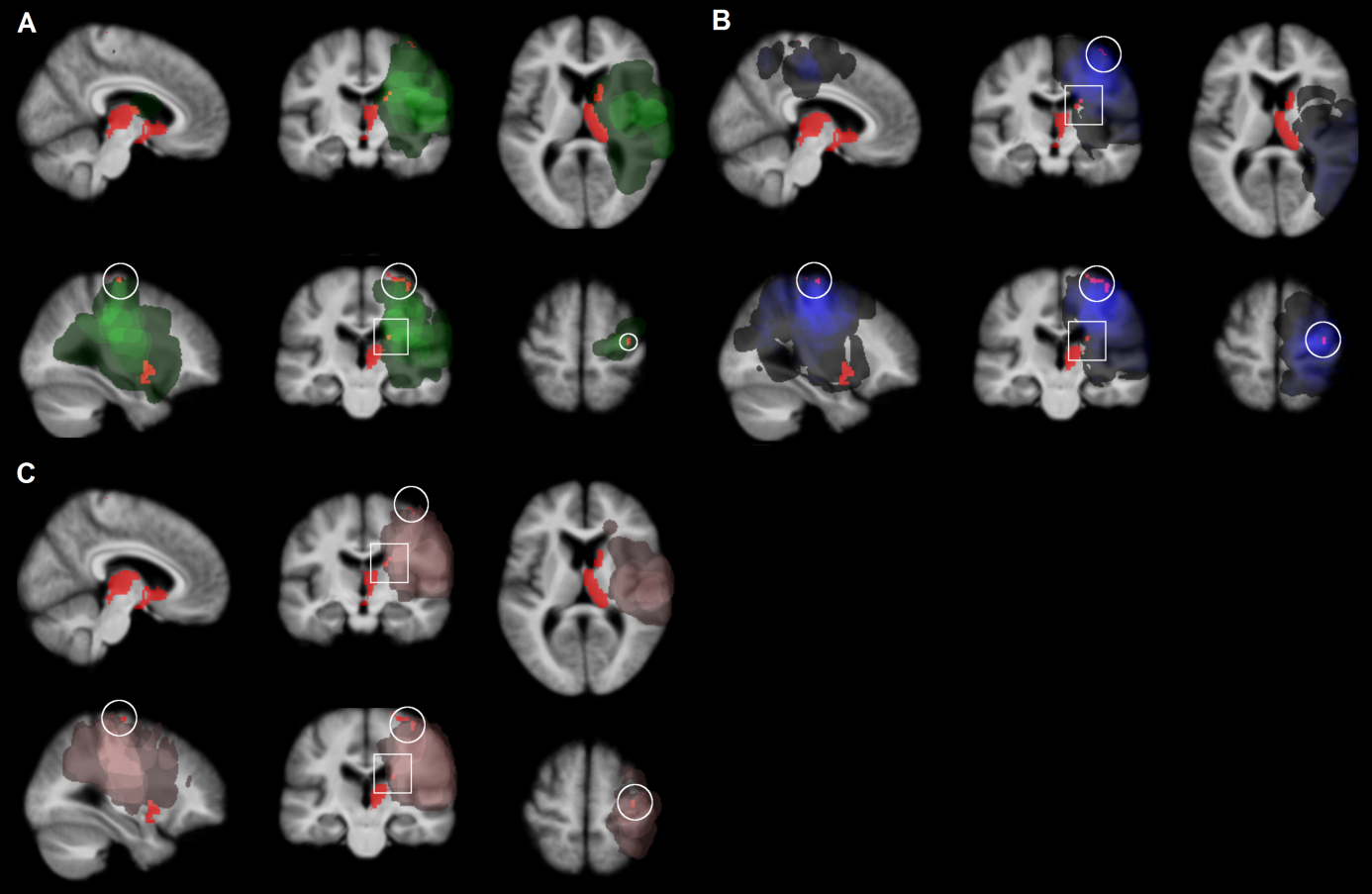


This figure illustrates the position of grey matter volume effects (red clusters) versus lesion summary maps of the fast (panel A), slow (panel B) and poor (panel C) recovery subgroups. White circle indicates the overlap between perilesional effect and corresponding lesion map, white square the overlap between subcortical effects and corresponding lesion map. Each row shows sagittal, coronal and axial slices centred on the subcortical (upper row coordinates : x, y, z = 8 ,-12 ,10) or perilesional effect (lower row coordinates: x ,y, z = 36, -20, 62). Images are displayed in neurological convention. Coordinates are in MNI space.

**Supplemental References**

Anderson DR, Burnham KP. Model selection and multi-model inference: A practical information-theoretic approach. New York: Springer; 2010.

Chestnut C, Haaland KY. Functional significance of ipsilesional motor deficits after unilateral stroke. Archives of physical medicine and rehabilitation. 2008 Jan;89(1):62-8.

Dyck PJ, O'Brien PC, Kosanke JL, Gillen DA, Karnes JL. A 4, 2, and 1 stepping algorithm for quick and accurate estimation of cutaneous sensation threshold. Neurology. 1993 Aug;43(8):1508-12.

Guttmann L. Some necessary conditions for common factor analysis. *Psychometrika*. 1954;19:149-61.

Jebsen RH, Taylor N, Trieschmann RB, Trotter MJ, Howard LA. An objective and standardized test of hand function. Archives of physical medicine and rehabilitation. 1969 Jun;50(6):311-9.

Mathiowetz V, Weber K, Volland G, Kashman N. Reliability and validity of grip and pinch strength evaluations. J Hand Surg Am. 1984 Mar;9(2):222-6.

Posada D, Buckley TR. Model selection and model averaging in phylogenetics: Advantages of akaike information criterion and bayesian approaches over likelihood ratio tests. Syst Biol. 2004 Oct;53(5):793-808.

Sunderland A. Recovery of ipsilateral dexterity after stroke. Stroke; a journal of cerebral circulation. 2000 Feb;31(2):430-3.

Sunderland A, Bowers MP, Sluman SM, Wilcock DJ, Ardron ME. Impaired dexterity of the ipsilateral hand after stroke and the relationship to cognitive deficit. Stroke; a journal of cerebral circulation. 1999 May;30(5):949-55.

Wagenmakers EJ, Farrell S. Aic model selection using akaike weights. Psychon Bull Rev. 2004 Feb;11(1):192-6.

Wetter S, Poole JL, Haaland KY. Functional implications of ipsilesional motor deficits after unilateral stroke. Archives of physical medicine and rehabilitation. 2005 Apr;86(4):776-81.
